# Electrophysiological Markers of *Ex-Situ* Heart Performance in a Porcine Model of Cardiac Donation After Circulatory Death

**DOI:** 10.3389/ti.2024.13279

**Published:** 2024-11-20

**Authors:** Jorik H. Amesz, Mark F. A. Bierhuizen, Sanne J. J. Langmuur, Paul Knops, Yvar P. van Steenis, Dwight Dumay, Mathijs S. van Schie, Olivier C. Manintveld, Natasja M. S. de Groot, Yannick J. H. J. Taverne

**Affiliations:** ^1^ Translational Cardiothoracic Surgery Research Lab, Department of Cardiothoracic Surgery, Erasmus University Medical Center, Rotterdam, Netherlands; ^2^ Translational Electrophysiology Lab, Department of Cardiology, Erasmus University Medical Center, Rotterdam, Netherlands; ^3^ Department of Cardiology, Erasmus University Medical Center, Rotterdam, Netherlands; ^4^ Erasmus MC Transplant Institute, Erasmus University Medical Center, Rotterdam, Netherlands; ^5^ Department of Clinical Perfusion, Erasmus University Medical Center, Rotterdam, Netherlands; ^6^ Department of Microelectronics, Faculty of Electrical Engineering, Mathematics and Computer Sciences, Circuits and Systems, Delft University of Technology, Delft, Netherlands

**Keywords:** cardiac transplantation, *ex situ* heart perfusion, machine perfusion, electrophysiological mapping, graft assessment

## Abstract

Normothermic *ex-situ* heart perfusion (ESHP) enables assessment of hearts donated after circulatory death (DCD) prior to transplantation. However, sensitive parameters of cardiac function of DCD hearts on ESHP are needed. This study proposes a novel approach using electrophysiological (EP) parameters derived from electrical mapping as biomarkers of post-ischemic cardiac performance. Porcine slaughterhouse hearts (PSH) were divided in two groups based on the type of warm ischemia (Group 1: 10 ± 1 min with animal depilation vs. Group 2: ≤5 min without depilation). Electrical mapping of the right (RV) and left ventricle (LV) was performed on ESHP. Potential voltages, slopes and conduction velocities were computed from unipolar electrograms and compared between groups. Voltages were lower in Group 1 compared to Group 2 (RV: 3.6 vs. 15.3 mV, p = 0.057; LV: 10.8 vs. 23.6 mV, p = 0.029). In addition, the percentage of low-voltage potentials was higher and potential slopes were flatter in Group 1. Voltages and slopes strongly correlated with the visual contractile performance of PSH, but showed weaker correlation with lactate profiles. In conclusion, unipolar potential voltages and potential slopes were decreased in hearts with severe warm ischemia. As such, EP parameters could aid transplantation teams in decision-making on transplantability of DCD hearts.

## Graphical Abstract

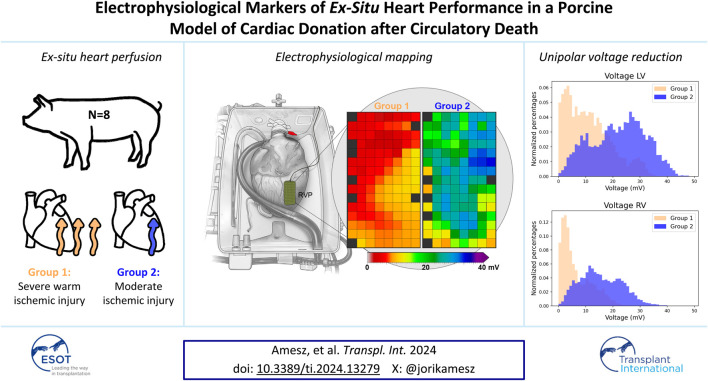

## Introduction

Normothermic *ex-situ* heart perfusion (ESHP) has been successful in enlarging the cardiac donor pool using circulatory death donors (DCD) [1–4]. Nonetheless, DCD hearts suffer from serious ischemia-reperfusion injury due to the inevitable functional warm ischemic time (WIT) prior to cardioplegic flush. For this reason, the post-ischemic cardiac quality needs to be assessed in a near-physiological beating state during ESHP.

Currently, quality assessment is based on lactate trends, together with measurements of coronary flow and aortic pressure, and visual contractile assessment of the heart by the transplantation surgeon. However, accuracy of lactate profiles for assessment of DCD hearts is subject of debate [5] and animal studies observed poor correlation between lactate levels and post-ischemic contractile function [6–8]. Additionally, lactate profiles could not reliably predict the need for mechanical circulatory support post-DCD heart transplantation in clinical practice [9]. Hence, additional, sensitive and real-time parameters of cardiac function are needed, especially in the setting of more marginal donor hearts. To date, novel sensitive biochemical markers have not been identified, and such markers need to be available as point-of-care test for eventual translation to clinical practice. Functional parameters, including ejection fraction, stroke volume and dP/dt have shown stronger correlations with myocardial performance [8], but cannot be assessed on the only clinical device that is currently available due to the Langendorff perfusion mode [10].

Electrical markers may provide rapid and real-time markers of ischemic damage. In daily clinical practice, the impact of myocardial ischemia on electrical function is detected by the surface electrocardiogram. Electrical mapping can also be used to detect effects of myocardial ischemia. This is defined as the recording of the summation of electrical activity of cardiomyocytes underlying mapping electrodes [11] and is performed directly on the epicardial surface of the donor heart on ESHP. As such, quantifiable electrophysiological (EP) parameters derived from local electrograms (EGMs) can be used to assess the electrical function of the ventricles and detect local regions of ischemia. Ion homeostasis is disturbed during ischemia [12] and does not return to pre-ischemic levels if damage from ischemia is severe [13]. Hence, donor hearts with severe injury from ischemia and reperfusion may present with impaired electrical function. To test this hypothesis, the purpose of this study was to investigate whether, and which, EP parameters are potential markers of cardiac tissue vitality and ischemic injury. Therefore, a high resolution electrical mapping approach was performed during ESHP on beating porcine slaughterhouse hearts (PSH) with good versus poor quality. The resulting objective EP parameters may aid the transplantation team in decision-making on transplantability of (marginal) donor hearts.

## Materials and Methods

### Porcine Heart Procurement

The current study was performed on PSH from animals (age: 6 months, weight: ∼95 kg) sacrificed for human consumption. The protocols in the abattoir were consistent with the EC regulations 1069/2009 regarding slaughterhouse animal material for research. The procurement protocol of PSH has been described before by others [14, 15], showing good contractile function and reproducible outcomes if hearts are procured before depilation of the animal’s skin [16]. However, hearts from PSH that undergo depilation in a dehairing machine are known to show poor cardiac function on ESHP [16]. To test our hypothesis, pigs were divided into two groups based on whether the heart was explanted before or after animal depilation.

The pigs were electrically stunned, hung and exsanguinated, during which 1.5–2 L of blood were collected and anticoagulation was immediately added (25,000 IU heparin, 2 mg Tirofiban). WIT was defined as the period from electrical stunning until start of cold cardioplegic flush. Group 1 had a longer WIT of approximately 10 min and animals were first washed in a dehairing machine of 60°C before excision of the thoracic organs via parasternal incision, as per standard protocol of the local abattoir. Group 2 had a shorter WIT of ≤5 min and the heart was procured before depilation of the pig, after which the thoracic cavity was closed and the animal continued in the standard abattoir process.

Hearts were immediately topologically cooled in 0.9% saline solution and the aorta was cannulated and clamped, in order to rapidly administer St Thomas Cardioplegic Solution (2 L with 2500 IU/L heparin) in the coronary arteries. Subsequently, hearts were transported on cold static storage to the laboratory (∼50-min drive). The period between administration of cardioplegia and reperfusion on ESHP was recorded as cold ischemic time.

### *Ex-Situ* Perfusion

Upon arrival at the lab, the ascending aorta and pulmonary artery (PA) were cannulated, caval veins ligated, and a vent was positioned in the left ventricle (LV), similar to the human ESHP transplantation protocol [10, 17]. A Langendorff perfusion system, consisting of a centrifugal pump, cardiotomy reservoir (Medtronic EL404) and oxygenator (Sorin Group Inspire 6F) was primed with a mixture of 500 mL gelofusin ((B. Braun, Melsungen, Germany) with 12.5 g mannitol, 250 mg methylprednisolone, 20 mEq sodium bicarbonate and 10,000 IU heparin) and 100 mL 20% albumin (Prothya Biosolutions, Netherlands B.V.). After priming, 1,500 mL of blood were added and the blood-priming solution mixture was heated to 35°C. The heart was reperfused by connecting the aortic cannula to the perfusion system, resulting in active coronary perfusion and spontaneous myocardial contractions. Cardioversion in case of ventricular fibrillation was accomplished by administration of 1.0 g of magnesium sulfate and electrical defibrillation. Perfusate exiting the PA cannula and LV vent was collected and recirculated. Adenosine (Adenocor, Sanofi) was infused at 0.12–0.60 mg/h to ensure perfusion with an aortic pressure of 75–85 mmHg and coronary flow of ≥750 mL/min. A maintenance solution (0.15 g/L calcium gluconate, 0.025 g/L magnesium sulfate, 0.11 g/L sodium chloride, 1.0 g/L glucose, 12.5 IU/L insulin (ASPART Sanofi, Paris, France)) was infused at 10–30 mL/h to compensate for the heart’s uptake of electrolytes and nutrients. Biventricular electrical stimulation was performed if the heart rate was <70 beats per minute.

### Blood Analysis

Arterial and venous blood samples were taken every 20 min to monitor electrolyte (Na^+^, K^+^, Ca^2+^), metabolite (glucose, bicarbonate, lactate) and blood gas (pH, pO_2_, pCO_2_) levels, and corrected accordingly. Measurements were performed with the i-STAT 1 analyzer (Abbott Point of Care Inc., Chicago, IL, United States) using CG4+ and CG8+ cartridges. Calcium concentrations were maintained low during initial reperfusion to reduce ischemia-reperfusion injury [18], and corrected to physiological levels afterwards. A baseline blood sample was measured before connection of the heart. Lactate trends were calculated as the slope of the difference in concentration between the first and last arterial sample after reperfusion.

### Epicardial Mapping

**FIGURE 1:**
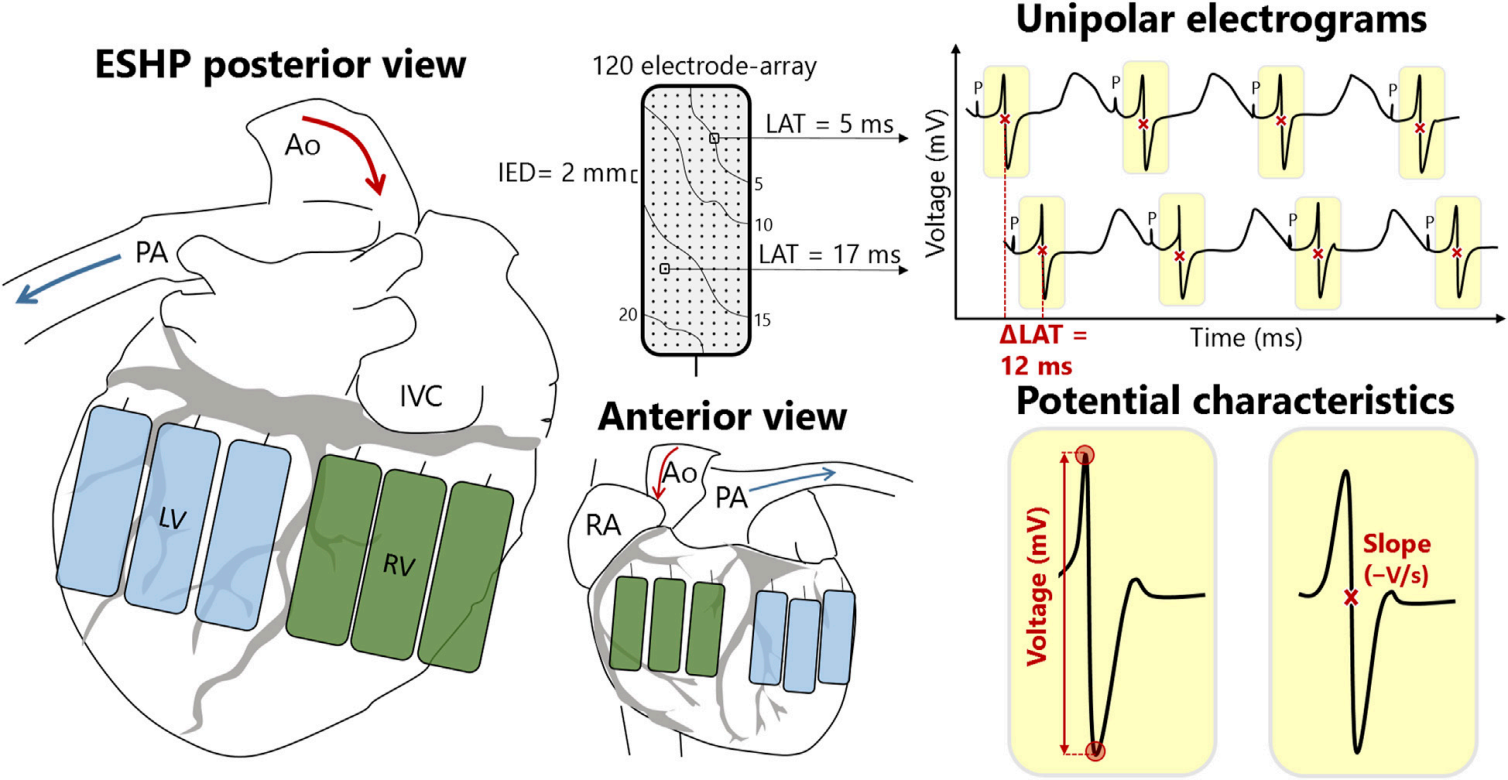
Schematic overview of right (RV) and left ventricular (LV) mapping locations and electrophysiological parameters. The rectangles represent the electrode array positioned at different locations at the LV (blue) and RV (green). Each mapping electrode consists of 120 electrodes that measure the electrical activity of the cardiomyocytes beneath the electrode. This results in 120 unipolar electrograms per mapping location, from which local activation times (LAT) and potential (voltage and slope) and conduction (velocity and block) characteristics can be calculated. *Ao*, aorta; *IED*, inter-electrode distance; *IVC*, inferior vena cava; *P*, pacing stimulus; *PA*, pulmonary artery; *RA*, right atrium.

High-resolution mapping of the heart was performed 60 min after reperfusion when the heart was stabilized on ESHP. The epicardial surface was systematically mapped by moving a custom-made 120-electrode array (electrode diameter 0.6 mm, inter-electrode distance 2 mm) over the RV and LV (Figure 1), comparable to our mapping approach during human cardiac surgery [19]. A steel wire was wrapped around the aorta as indifferent electrode. Five seconds were recorded at every mapping position, with a total recording time of approximately 5 min.

Recordings were sampled with a rate of 2 kHz, filtered with bandwidth 0–500 Hz, analog-to-digital converted (16 bits) and stored on hard disk. Color-coded local activation time (LAT) maps were created by annotating the steepest negative deflection of each unipolar potential to study abnormalities in myocardial conduction [19]. Conduction block was determined as a difference in conduction time of ≥12 ms between two adjacent electrodes and the prevalence of conduction block was calculated as percentage of all conduction times. Local effective conduction velocity (CV) was computed from LATs of neighboring electrodes using discrete velocity vectors [20]. In addition, color-coded maps were created visualizing unipolar extracellular potential voltages (peak-to-peak amplitudes) and slopes at each electrode [19]. Low-voltage was defined as the 5^th^ percentile of all peak-to-peak amplitudes from the RV and LV respectively, and rounded to the nearest integer.

### Contractile Assessment

Videos were recorded of all hearts and three transplant surgeons of the Erasmus MC who use ESHP in clinical practice were asked to independently assess visual contractility of the hearts. The surgeons were blinded for group assignment. They scored contractility of the whole heart, LV and RV on a scale of 1–5. In addition, they judged whether hearts were suitable for transplantation based on visual contractile performance and hearts were deemed suitable for transplantation if at least 2 surgeons agreed.

### Data Analysis

Median unipolar potential voltage, potential slope, and conduction velocity and the amount of low-voltage potentials and conduction block were calculated from the EGMs of the LV and RV from each PSH. LV and RV data were separated because of intrinsic differences in electrophysiological properties between both chambers [21], and the fact that the LV is unloaded during Langendorff perfusion. For these EP parameters, a median was calculated as a summary measure per PSH. These data were then presented as median (range) per group and a Mann-Whitney U test was performed to test for differences between groups. Blood gas and contractile data were tested for normality using QQ-plots and a Shapiro-Wilk test. Continuous variables were presented as mean ± standard deviation if normally distributed and as median (range) otherwise. Categorical variables were reported as number (percentage). An unpaired t-test or Mann-Whitney U test was performed, as appropriate. A p-value of ≤0.05 was considered statistically significant. A Pearson correlation test was performed to assess the degree of correlation between lactate, visual contractile assessment, and EP parameters. A correlation coefficient of <0.3 was considered as weak correlation, ≥0.3 – <0.7 as moderate correlation, and ≥0.7 as strong correlation. One-way analysis of variance testing was performed to test for differences across different ventricular regions of the heart. Statistical testing was performed using SPSS software (version 28.0.1.0 (142)). Line plots were created using Graphpad Prism (version 10.2.3 (403)). Correlation plots were created using R (version 4.3.1).

## Results

**FIGURE 2:**
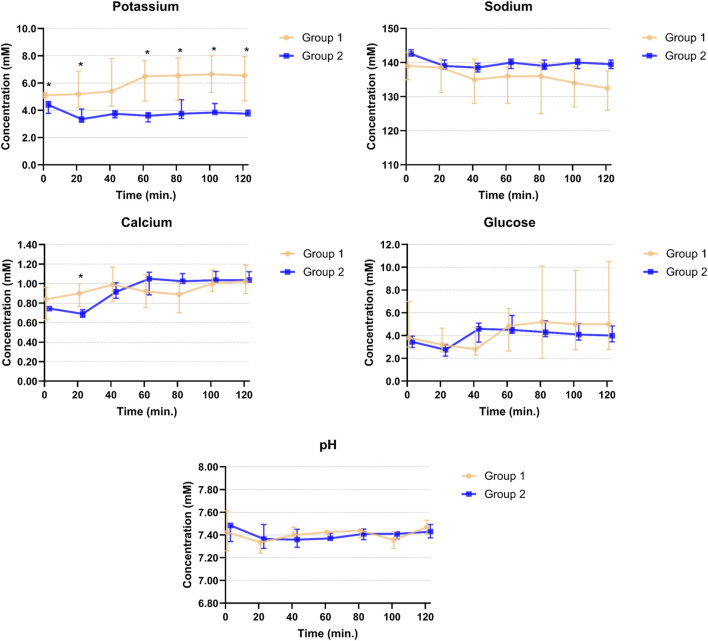
Potassium, sodium, calcium, glucose and pH levels in venous perfusate samples of Groups 1 and 2 during 2 hours of perfusion. Points are plotted as median values with interquartile ranges and asterisks mark significant differences between the two groups.

All hearts (n = 8) were successfully resuscitated and cold ischemic times were similar in Group 1 (n = 4) and Group 2 (n = 4) (104 ± 5 min vs. 111 ± 6 min, p = 0.157). All hearts required defibrillation after reperfusion, but hearts in Group 2 were easier to restart. Biventricular electrical stimulation was performed in all hearts to compensate for low heart rates, but 2 hearts in Group 2 failed to capture LV pacing resulting in only RV pacing. The blood levels of electrolytes, glucose and pH during 2 h of perfusion are presented in Figure 2. Sodium, calcium and glucose concentrations, and pH were similar between groups. Low calcium levels were corrected earlier in Group 1, but concentrations were similar after 1 h of perfusion. Potassium concentrations were significantly higher in Group 1, compared to Group 2 (Figure 2).

**FIGURE 3:**
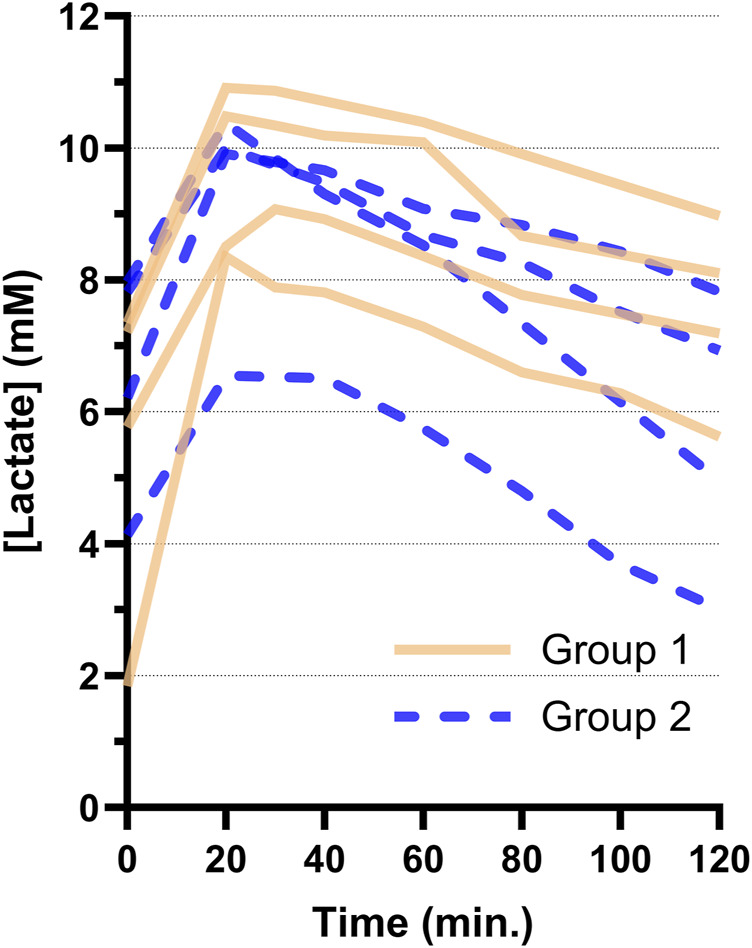
Arterial lactate trends per heart during 2 hours of *ex-situ* perfusion for Groups 1 and 2. The first sample after connection and reperfusion of the heart was taken at 20 min. All hearts showed a decreasing lactate trend after reperfusion.

All hearts showed a decreasing lactate trend after start of ESHP (Figure 3) without statistically significant difference between Groups 1 and 2 (−24 ± 6 μM/min vs. −36 ± 14 μM/min, p = 0.173). In addition, lactate concentrations (Group 1: 9.0 ± 1.5 mM vs. Group 2: 8.0 ± 1.5 mM, p = 0.371) and arteriovenous differences in lactate concentration (Supplementary Table S3) were not different between groups at the time of electrical mapping.

### Electrical Mapping

Electrical mapping on the RV and LV resulted in a total of >69,000 unipolar potentials. The data per individual heart were provided in Supplementary Tables S1, S2. Unipolar potential voltage was lower in Group 1 compared to Group 2, for both the RV (3.6 mV(2.5–12.8) vs. 15.3 mV (11.8–17.0), p = 0.057) and LV (10.8 mV(4.7–17.8) vs. 23.6 mV(19.4–24.2), p = 0.029) (Table 1). In addition, the median percentage of low-voltage potentials (RV: 1 mV, LV: 2 mV) was higher in Group 1 (RV: 10.6% (0.3–17.2) vs. 0.3% (0.1–0.5), p = 0.114; LV: 7.7% (0.6–23.0) vs. 0.4% (0.1–1.8), p = 0.200). Also, slopes of potentials were lower in Group 1 (RV: −0.2 V/s (−0.1 to −1.0) vs. −1.2 V/s (−0.5 to −1.4), p = 0.057; LV: −0.3 V/s (−0.2 to −1.8) vs. −1.1 V/s (−1.1 to −1.6), p = 0.343) but these differences did not reach statistical significance. Median conduction velocity and amount of conduction block did not differ between groups (Table 1).

**TABLE 1:** Outcomes of electrophysiological parameters measured 60 min after reperfusion between two groups with different types of ischemia. Data expressed as median (range).

	Group 1 (N = 4)	Group 2 (N = 4)	P-value
*Right ventricle*			
Number of potentials	3,736 (3,142–5,016)	3,045 (2,628–4,854)	0.343
Voltage (mV)	3.6 (2.5–12.8)	15.3 (11.8–17.0)	0.057
Low voltage (%) <1.0 mV	10.6 (0.3–17.2)	0.3 (0.1–0.5)	0.114
Slope (−V/s)	0.2 (0.1–1.0)	1.2 (0.5–1.4)	0.057
Conduction velocity (cm/s)	78.7 (32.1–94.5)	82.3 (80.1–86.0)	1.000
Conduction block (%)	10.3 (2.9–15.6)	6.2 (4.3–14.9)	0.686
*Left ventricle*			
Number of potentials	4,923 (3,942–5,588)	5,402 (4,182–5,825)	0.486
Voltage (mV)	10.8 (4.7–17.8)	23.6 (19.4–24.2)	**0.029**
Low voltage (%) <2.0 mV	7.7 (0.6–23.0)	0.4 (0.1–1.8)	0.200
Slope (−V/s)	0.3 (0.2–1.8)	1.1 (1.1–1.6)	0.343
Conduction velocity (cm/s)	90.2 (26.4–105.3)	89.3 (74.6–93.0)	0.886
Conduction block (%)	8.9 (4.0–19.0)	4.8 (3.1–5.4)	0.343

**FIGURE 4:**
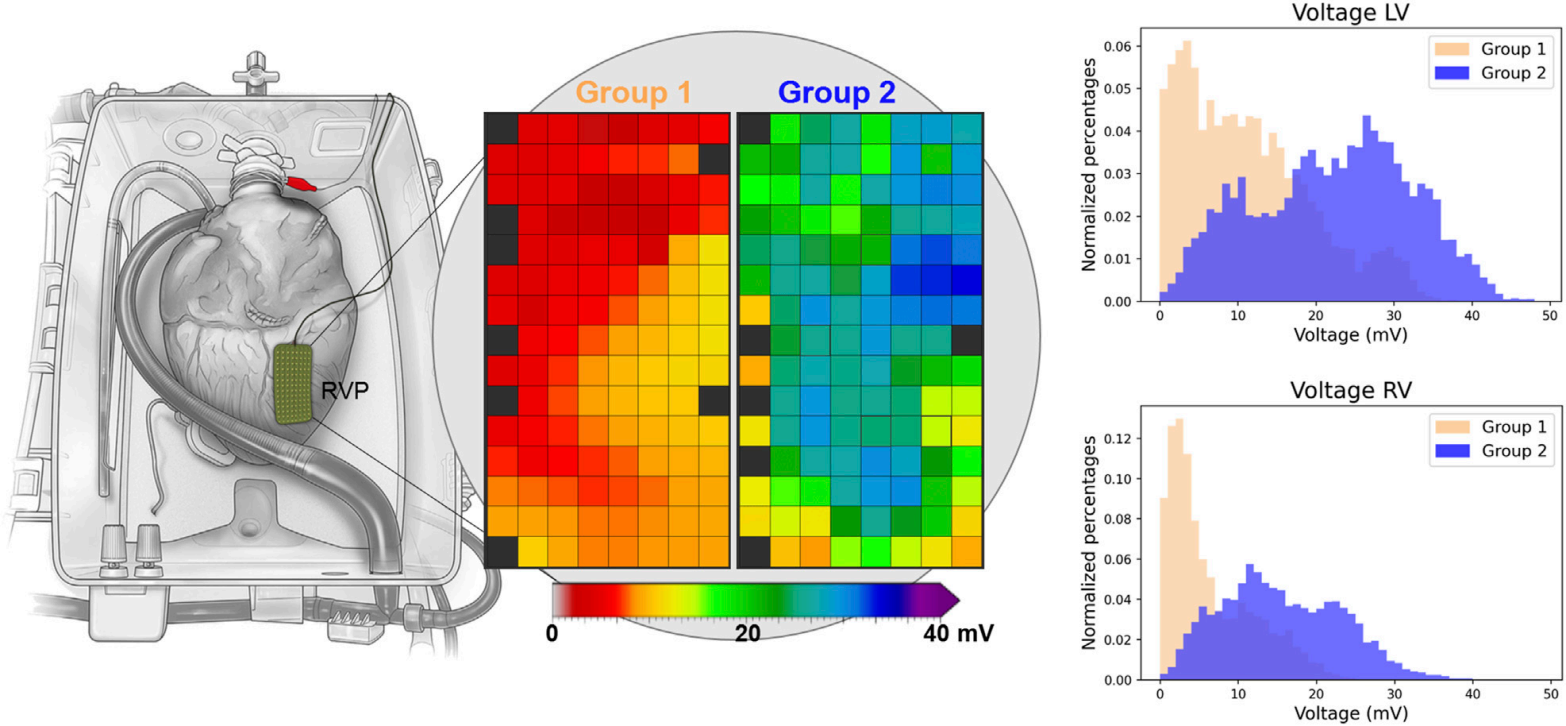
Exemplary voltage maps of Groups 1 and 2 of a mapping position on the right ventricular posterior (RVP) wall. Red and orange colours indicate lower voltages in Group 1. The histograms of all deflections of the RV (*n* = 29,201) and LV (*n* = 34,626) show lower voltages in Group 1. “Reprinted from The Lancet, Vol 385, Ardehali et al. [10]; Figure 1D, Copyright 2015, with permission from Elsevier.”

Figure 4 presents exemplary colour-coded voltage maps of the posterior wall of the RV from both groups. The red/orange colours in Group 1 indicate lower potential voltages, compared to higher potential voltages represented by green/blue colours in Group 2. In addition, histograms showed lower voltages in Group 1 (Figure 4). Differences between the anterior, lateral and posterior regions of the ventricles are represented in Supplementary Figure S1, without a clear preferential location for lower potential voltages.

### Contractile Performance

Cardiac visual contractile performance was scored 1.5 ± 0.5 in Group 1 and 3.0 ± 1.0 in Group 2 (p = 0.042), thus showing worse cardiac function in the hearts procured after depilation. All hearts in Group 1 were deemed unsuitable for transplantation unanimously, and 2/4 hearts in Group 2 were considered transplantable. Exemplary videos of contractile performance of PSH in both groups are presented in the Supplementary Material.

### Correlation

**FIGURE 5:**
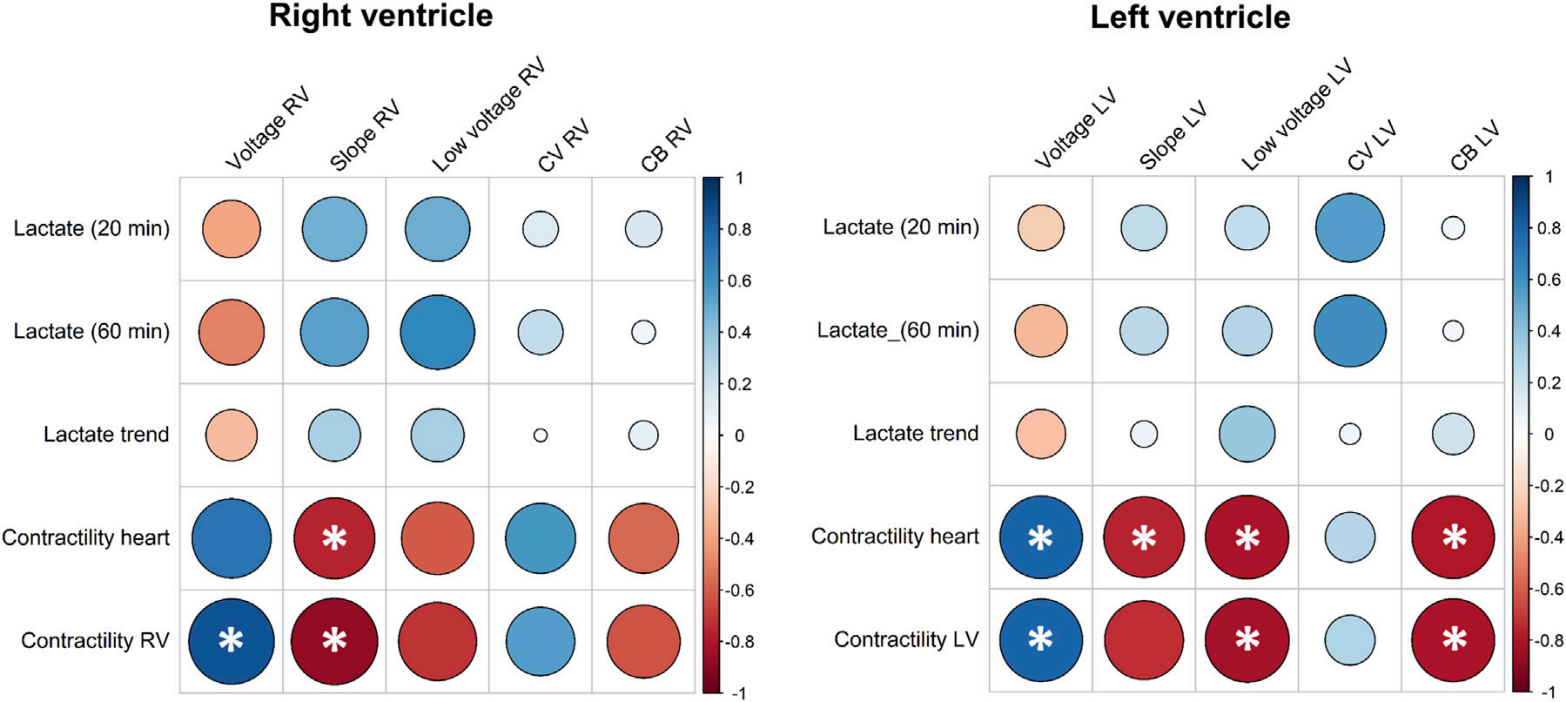
Pearson correlation coefficients between arterial lactate concentrations after reperfusion (20 min) and time of electrical mapping (60 min), arterial lactate trend, visual contractile performance, and electrophysiological parameters of the RV and LV. Larger circles indicate stronger correlation coefficients and asterisks indicate statistical significance at the 0.05 level. *CB*, conduction block; *CV*, conduction velocity; *LV*, left ventricle; *RV*, right ventricle.

Lactate levels and visual contractile assessment were correlated to EP characteristics of the RV and LV (Figure 5; Supplementary Figure S2). Weak to moderate correlations were observed between lactate and EP parameters (Figure 5). In comparison, correlations were stronger between EP parameters and contractility of the RV and LV (Figure 5). Median potential voltage of the RV significantly correlated with the contractility of the RV and median potential slopes significantly correlated with the visual contractile performance of the heart and RV. For the LV, strong significant correlations were observed between the visual contractile performance and potential voltages, slopes, low-voltage and amount of conduction block (Figure 5). Supplementary Figure S2 also showed a significant correlation between the lactate trend and visual contractile performance of the heart and LV, but not with the lactate levels at start and 1 hour after reperfusion.

## Discussion

### Key Findings

This is the first study to measure and identify EP markers for the assessment of graft quality of hearts preserved on normothermic ESHP for cardiac transplantation. Unipolar potential voltages and potential slopes were decreased in hearts with extensive ischemic injury in a porcine DCD model. Voltage measured from unipolar EGMs might be an additional marker of graft viability. As such, EP parameters assessed by high-resolution epicardial mapping could serve as valuable bioelectrical markers, in addition to other metabolic and hemodynamic parameters, of the functional status of DCD hearts on ESHP.

### Lactate Metabolism

The lactate levels of PSH in the current study all showed a decreasing trend, which makes it impossible to distinguish which hearts were suitable for transplantation based on these curves (Figure 3). Furthermore, contractile performance of certain hearts was very poor (Supplementary Video S1) and these hearts were indisputably rejected for transplantation. As such, a decreasing lactate trend alone was not a sensitive marker of graft performance in our PSH model of DCD ESHP. This is in accordance with animal studies [6–8] and clinical outcomes [9].

### Hyperkalemia

Potassium concentrations were significantly higher in Group 1 compared to Group 2. Hyperkalemia was associated with reduced cardiac function in other ESHP studies [22, 23] and is believed to be the result of prolonged ischemia [24–26] and the slaughtering and dehairing in the abattoir [14]. An acute decrease in pH due to electrical stunning during the slaughter process as previously reported [14] was not observed in this study. Hyperkalemia causes electrical changes in the cardiac action potential (increased resting potential, slower upstroke, and reduced duration) [26, 27] which is reflected in the EGM morphology. An increase in resting potential due to hyperkalemia might contribute to the observed decrease in potential voltage in our study [24]. A reduced conduction velocity as expected in hyperkalemic conditions was not observed in Group 1 [27]. We contend that cardiac mapping can offer additional insights beyond perfusate potassium measurements alone, particularly since electrogram changes due to tissue damage may still be detected even after hyperkalemia is resolved.

### Electrophysiological Markers of Ischemic Damage

Lower RV and LV unipolar potential voltages were observed in Group 1, compared Group 2 (Table 1). In addition, a clear difference in the number of low-voltage potentials was observed between both groups, although not reaching statistical significance due to relatively high voltages in one heart in Group 1 (Supplementary Tables S1, S2). Next, unipolar voltage and the amount of low-voltage potentials were strongly correlated to the contractility of the heart, especially the LV. As such, a lower unipolar potential voltage corresponds with more injury from ischemia and reperfusion. Likewise, low-voltages of <1.0 mV for the RV and <2.0 mV for the LV seem to be a sensitive marker of severe injury caused by ischemia and reperfusion.

Such reduction in potential voltage following acute ischemia (and reperfusion) was previously demonstrated in animal experiments investigating the effects of coronary artery occlusions [28, 29] and humans [30]. Additionally, potential voltage is also known to be reduced in regions affected by myocardial infarction [31, 32] where low voltage potentials may indicate scarred myocardium in which the metabolism is reduced and irreversibly damaged [32]. Hence, the current study demonstrates that measurements of unipolar potential voltages on DCD hearts are valuable to assess the amount of irreversible myocardial injury caused by warm ischemia.

Similarly, slowing of conduction following coronary occlusion is a well-known phenomenon [33], potentially leading to post-infarction ventricular tachycardias [34, 35]. However, we did not observe more conduction disorders in Group 1. Yet, conduction velocity and conduction block seem to be less sensitive markers of ischemia-reperfusion injury compared to potential voltage, at least in the acute setting of DCD donation and subsequent reperfusion with ESHP. Nevertheless, the advantage of our mapping approach is that it quickly provides multiple EP parameters which could serve as valuable adjunct in graft quality assessment. These features can be easily visualized using color-coded voltage and conduction maps (Figure 4), which makes it possible to detect local regions of ischemic damage in the DCD heart, whereas lactate levels only reflect the total performance of the donor heart.

### Limitations

The cold ischemic time in our study was longer than the usual duration for ESHP machine setup in clinical practice because of the transport time from the abattoir to the laboratory. In addition, the study was performed on slaughterhouse animals, which do not allow for easy control of cardiac explantation conditions as compared to laboratory animals. Animals in Group 1 were exposed to skin depilation before cardiac explantation, which does not resemble a clinical DCD scenario. Conversely, models using coronary ligation to produce myocardial ischemia do not reflect global ischemia from DCD procedures [36]. Depilation is known to consistently reduce cardiac function on ESHP [16] and can therefore be considered a reasonable alternative to induce global ischemia, despite being a little drastic. In our study, EP markers were isolated for functional assessment of hearts on ESHP, which should be further validated in a more clinical setting.

Another limitation is the sample size which reduces the study’s power and may overstate the significance of findings. No correction for multiple testing was performed, which could have introduced the possibility of type I errors. The reported p-values are intended to provide an indication of potential differences between groups, rather than conclusive evidence of significant effects.

Furthermore, EP parameters were only measured 60 min after reperfusion and the experiment only lasted 2 h, which is shorter than the duration for which hearts are typically perfused in the clinic. Yet, measurements at multiple time points on ESHP might possibly provide more insights in the effects of ischemia-reperfusion injury on myocardial survival. Additionally, contractile performance was assessed visually, which resembles the current clinical practice, but lacks the precision and rigor of more objective methods, e.g., pressure catheters [5, 37] or ventricular balloons [38].

### Future Perspectives

As a next step, evaluation of the approach of this study on human hearts transported on ESHP for cardiac transplantation is essential to identify reference values differentiating between healthy and injured tissue [39]. Consequently, electrical mapping and other novel assessment strategies might push the boundaries of ischemic time and donor criteria to further enlarge the cardiac donor pool in the future.

### Conclusion

EP parameters derived from electrical mapping can aid in decision-making on transplantability of donor hearts transported on ESHP. Low-voltage potentials might be a sensitive marker of myocardial ischemic damage and can be easily combined with other EP markers including myocardial conduction characteristics. Furthermore, electrical mapping can detect local regions of ischemia and the output is visualized in a user-friendly manner to the transplantation team. As such, the presented technique introduces novel, objective EP biomarkers in addition to lactate profiles and visual contractile assessments, and may serve as novel additional diagnostic procedure for assessing graft function, especially in marginal donors.

## Data Availability

The datasets presented in this article will be made available upon reasonable request to the corresponding author. Requests to access the datasets should be directed to y.j.h.j.taverne@erasmusmc.nl.
