# Supplementary material for: Electrophysiological Markers of Ex-Situ Heart Performance in a Porcine Model of Cardiac Donation After Circulatory Death
Source: Transpl Int. 2024 Nov 20;37:13279. doi: 10.3389/ti.2024.13279 (PMC11616589; doi:10.3389/ti.2024.13279)
Supplement: Supplementary file 1 [file Presentation1.pptx]

## Slide 1
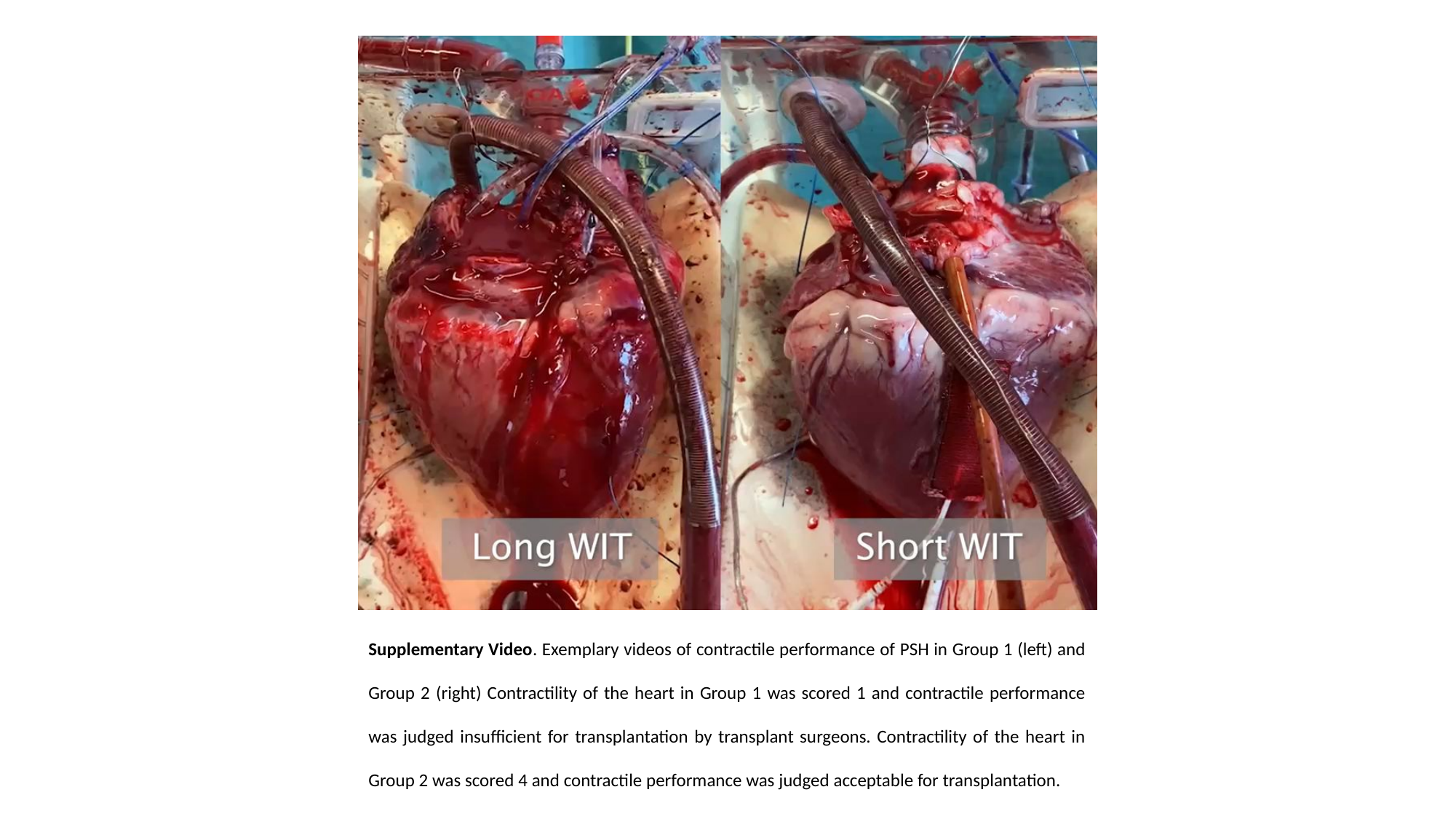

Supplementary Video. Exemplary videos of contractile performance of PSH in Group 1 (left) and Group 2 (right) Contractility of the heart in Group 1 was scored 1 and contractile performance was judged insufficient for transplantation by transplant surgeons. Contractility of the heart in Group 2 was scored 4 and contractile performance was judged acceptable for transplantation.
