# Supplementary material for: Electrophysiological Markers of Ex-Situ Heart Performance in a Porcine Model of Cardiac Donation After Circulatory Death
Source: Transpl Int. 2024 Nov 20;37:13279. doi: 10.3389/ti.2024.13279 (PMC11616589; doi:10.3389/ti.2024.13279)
Supplement: Supplementary file 2 [file DataSheet1.docx]

**Supporting Information
Electrophysiological Markers of Ex-Situ Heart Performance in a Porcine Model of Cardiac Donation after Circulatory Death**

Jorik H. Amesz, Mark F.A. Bierhuizen, Sanne J.J. Langmuur, Paul Knops, Yvar P. van Steenis, Dwight Dumay, Mathijs S. van Schie, Olivier C. Manintveld, Natasja M.S. de Groot, Yannick J.H.J. Taverne

- **Supplementary Table 1**
- **Supplementary Table 2**
- **Supplementary Table 3**
- **Supplementary Figure 1**
- **Supplementary Figure 2**

**Supplementary Table 1.** Electrophysiological data of the right ventricle per heart of Groups 1 and 2.

| **Name** | **N** | **Voltage (mV)** | **Low voltage (%)** | **Slope (−V/s)** | **Conduction velocity (cm/s)** | **Conduction block (%)** |
| --- | --- | --- | --- | --- | --- | --- |
| **Group 1: Heart A** | 3142 | 4.24 | 5.79 | -0.13 | 32.10 | 15.59 |
| **Group 1: Heart B** | 3661 | 3.01 | 17.15 | -0.17 | 94.48 | 5.83 |
| **Group 1: Heart C** | 5016 | 12.77 | 0.26 | -1.02 | 87.44 | 2.90 |
| **Group 1: Heart D** | 3811 | 2.54 | 15.46 | -0.13 | 69.96 | 14.95 |
| **Group 2: Heart A** | 3206 | 16.24 | 0.22 | -1.43 | 85.97 | 6.72 |
| **Group 2: Heart B** | 2628 | 16.99 | 0.53 | -1.23 | 83.59 | 4.34 |
| **Group 2: Heart C** | 2883 | 11.81 | 0.42 | -0.54 | 80.96 | 14.91 |
| **Group 2: Heart D** | 4854 | 14.38 | 0.10 | -1.09 | 80.08 | 5.62 |

**Supplementary Table 2.** Electrophysiological data of the left ventricle per heart of Groups 1 and 2.

| **Name** | **N** | **Voltage (mV)** | **Low voltage (%)** | **Slope (−V/s)** | **Conduction velocity (cm/s)** | **Conduction block (%)** |
| --- | --- | --- | --- | --- | --- | --- |
| **Group 1: Heart A** | 4791 | 5.76 | 14.65 | 0.17 | 26.35 | 18.98 |
| **Group 1: Heart B** | 3942 | 15.86 | 0.56 | 0.46 | 105.31 | 5.35 |
| **Group 1: Heart C** | 5055 | 17.81 | 0.69 | 1.77 | 87.00 | 3.97 |
| **Group 1: Heart D** | 5588 | 4.71 | 23.01 | 0.20 | 93.38 | 12.49 |
| **Group 2: Heart A** | 5243 | 24.17 | 0.29 | 1.15 | 74.62 | 3.12 |
| **Group 2: Heart B** | 4182 | 24.15 | 1.84 | 1.12 | 87.84 | 5.39 |
| **Group 2: Heart C** | 5825 | 23.05 | 0.14 | 1.56 | 93.00 | 4.47 |
| **Group 2: Heart D** | 5561 | 19.35 | 0.58 | 1.11 | 90.80 | 5.03 |

**Supplementary Table 3.** Arterial and venous lactate concentrations and arteriovenous difference in lactate concentration at time of mapping per heart of Groups 1 and 2.

| **Name** | **[Lactate]_art._ (mM)** | **[Lactate]_ven._ (mM)** | **[Lactate]_art.−ven._ (mM)** |
| --- | --- | --- | --- |
| **Group 1: Heart A** | 7.29 | 7.75 | -0.46 |
| **Group 1: Heart B** | 10.09 | 10.12 | -0.03 |
| **Group 1: Heart C** | 8.36 | 8.04 | 0.32 |
| **Group 1: Heart D** | 10.39 | 10.14 | 0.25 |
| **Group 2: Heart A** | 5.75 | 5.59 | 0.16 |
| **Group 2: Heart B** | 9.08 | 8.91 | 0.17 |
| **Group 2: Heart C** | 8.68 | 8.56 | 0.12 |
| **Group 2: Heart D** | 8.53 | 8.31 | 0.22 |

**
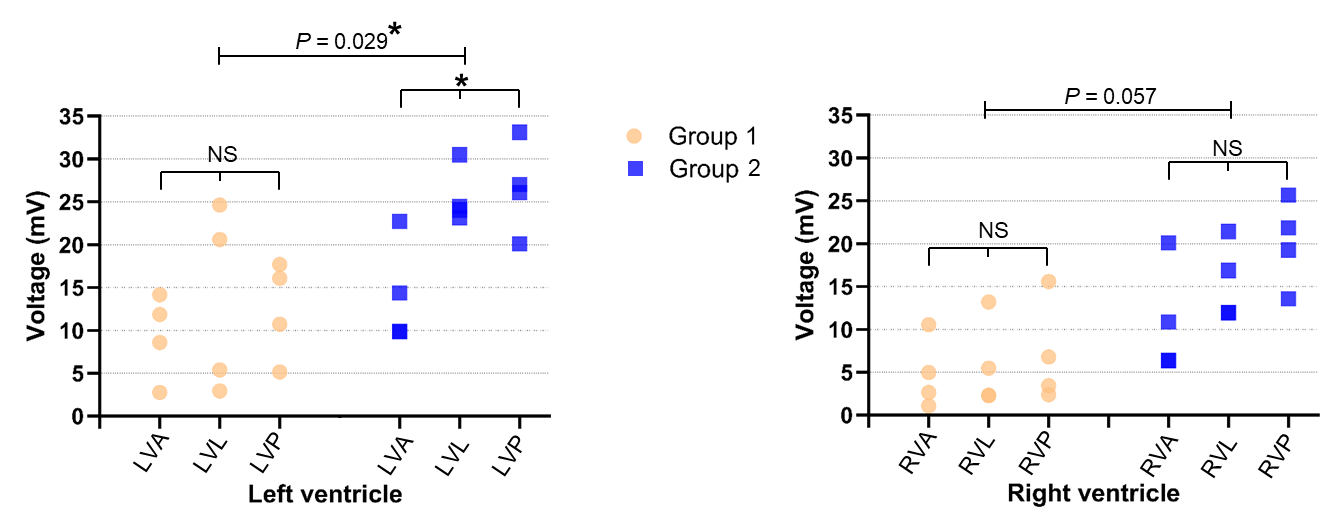
**

**Supplementary Figure 1.** Dot plots represent the voltage distribution of each PSH across the posterior, lateral and anterior wall of the right (RVP, RVL, RVA) and left ventricle (LVP, LVL, LVA).


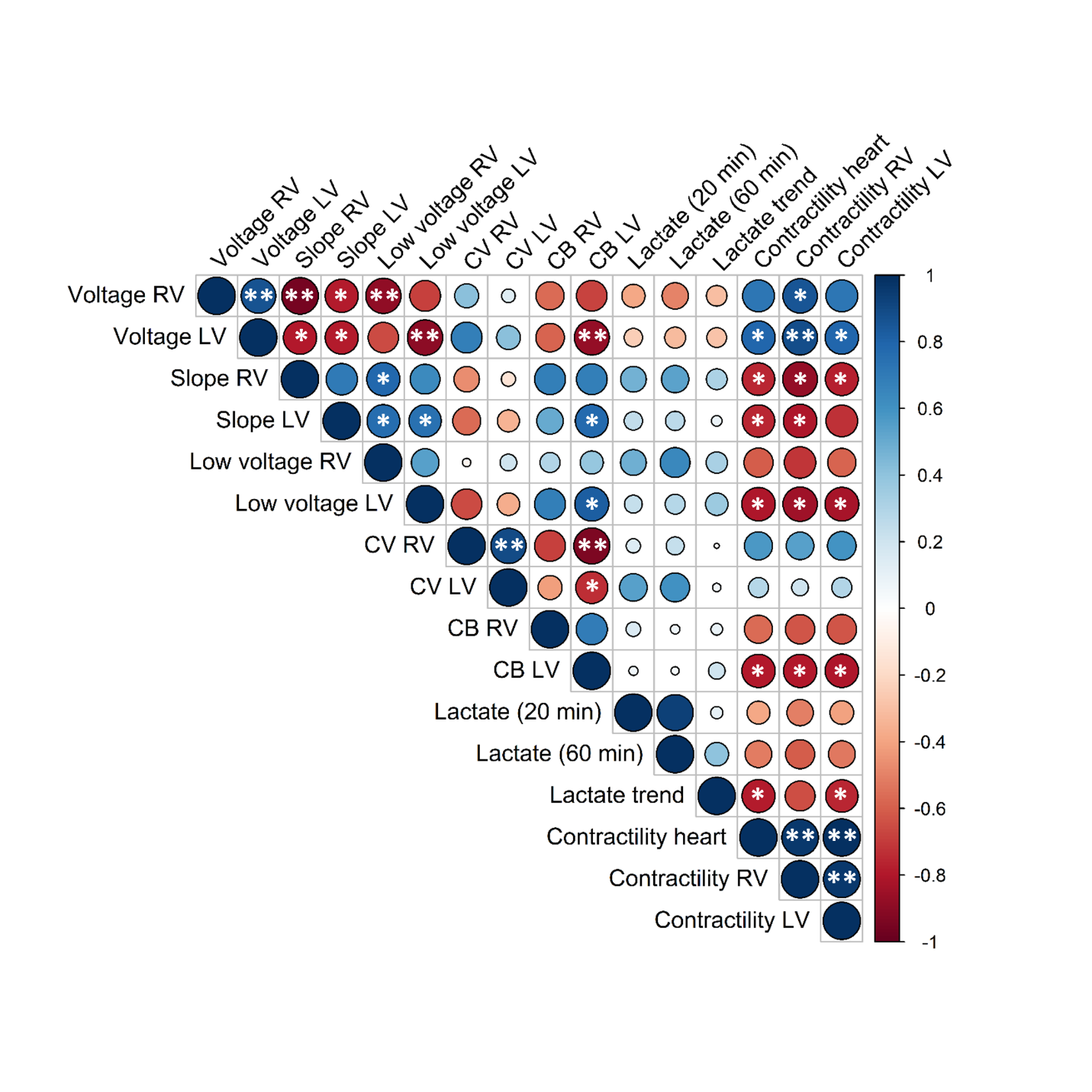


**Supplementary Figure 2.** Correlation plot of lactate, visual contractile performance and electrophysiological parameters. * p≤0.05, ** p≤0.001.

*CB* = conduction block. *CV* = conduction velocity. *LV* = left ventricle. *RV* = right ventricle.
